# Supplementary material for: Comprehensive Annotation and Functional Exploration of MicroRNAs in Lettuce
Source: Front Plant Sci. 2021 Dec 24;12:781836. doi: 10.3389/fpls.2021.781836 (PMC8739914; doi:10.3389/fpls.2021.781836)
Supplement: Supplementary file 2 [file Data_Sheet_8.ZIP › GSM2230748.plot/Lsa-miR159a_Lsat_1_v5_gn_5_34141.1_503_TPlot.pdf]

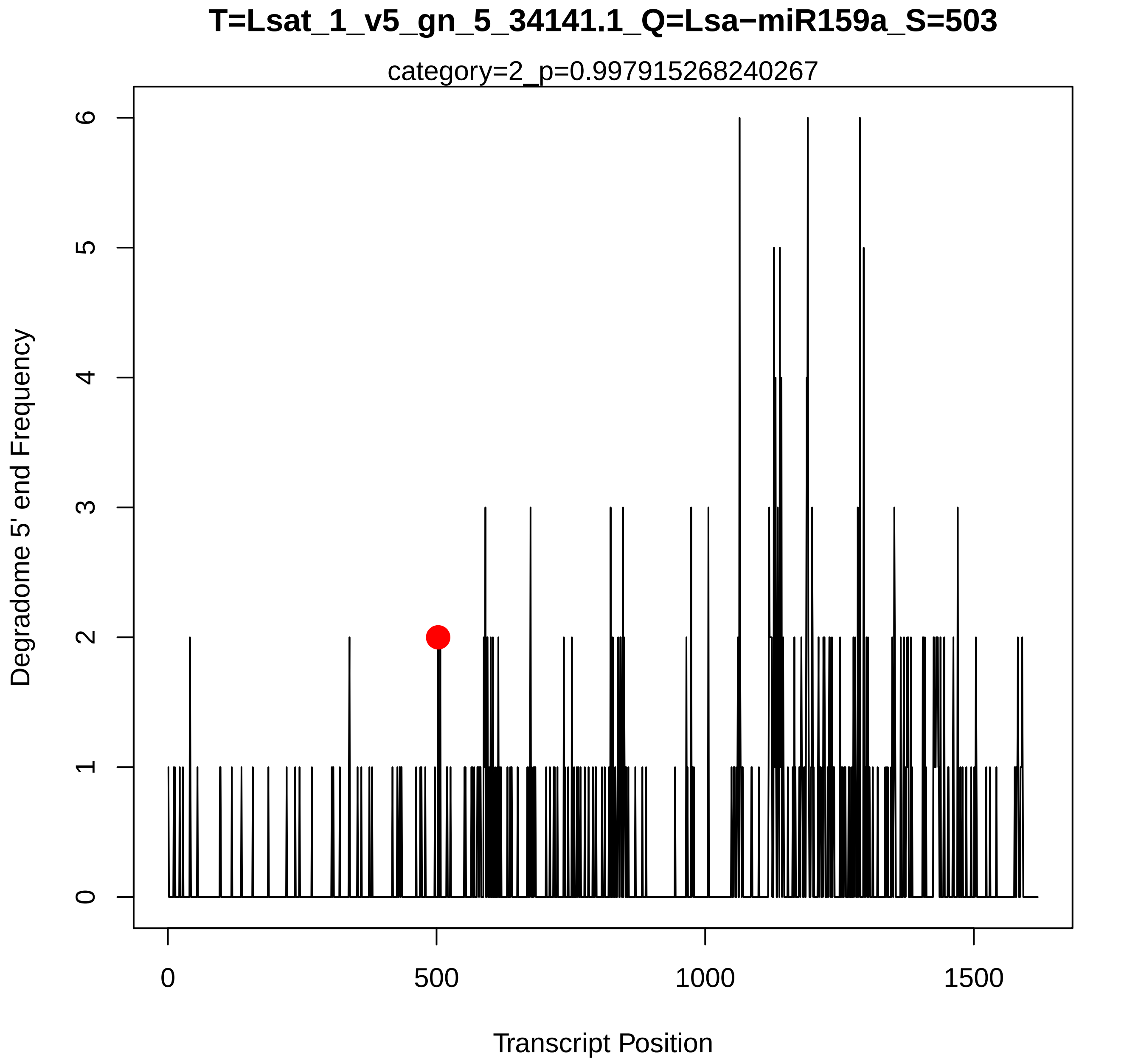
T=Lsat_1_v5_gn_5_34141.1_Q=Lsa−miR159a_S=503
category=2_p=0.997915268240267
Degradome 5' end Frequency
0
1
2
3
4
5
6
Transcript Position
0
500
1000
1500
